# Exome sequencing identifies *PD‐L2* as a potential predisposition gene for lymphoma

**DOI:** 10.1002/hon.3033

**Published:** 2022-05-29

**Authors:** Jianming Shao, Lei Gao, Marco L. Leung, Bailey Gallinger, Cara Inglese, M. Stephen Meyn, Daniela Del Gaudio, Soma Das, Zejuan Li

**Affiliations:** ^1^ Department of Pathology and Genomic Medicine Houston Methodist Hospital Houston Texas USA; ^2^ Department of Human Genetics The University of Chicago Chicago Illinois USA; ^3^ The Steve and Cindy Rasmussen Institute for Genomic Medicine Nationwide Children's Hospital Columbus Ohio USA; ^4^ Departments of Pathology and Pediatrics The Ohio State University College of Medicine Columbus Ohio USA; ^5^ Cancer Genetics Program The Hospital for Sick Children Toronto Ontario Canada; ^6^ Center for Human Genomics and Precision Medicine University of Wisconsin Madison Wisconsin USA

**Keywords:** germline predisposition, lymphoma, *PD‐L2*

## Abstract

To investigate germline predisposition in lymphoma, we performed whole‐exome sequencing and discovered a novel variant (c.817‐1G>T) in programmed cell death 1 ligand 2 (*PD‐L2*) in a family with early‐onset lymphomas and other cancers. The variant was present in the proband with follicular lymphoma and his son with Hodgkin's lymphoma. It was in the terminal splice acceptor site of *PD‐L2* and embedded in a putative enhancer of Janus kinase 2 (*JAK2*) and programmed cell death 1 ligand (*PD‐L1*). We also found that gene expression of *PD‐L2*, *PD‐L1*, and *JAK2* was significantly increased. Using 3′ rapid amplification of cDNA ends (3′ RACE), we detected an abnormal *PD‐L2* transcript in the son. Thus, the c.817‐1G>T variant may result in the elevated *PD‐L2* expression due to the abnormal *PD‐L2* transcript and the elevated *PD‐L1* and *JAK2* expression due to increased enhancer activity of *PD‐L1* and *JAK2*. The *PD‐L2* novel variant likely underlies the genetic etiology of the lymphomas in the family. As *PD‐L2* plays critical roles in tumor immunity, identification of *PD‐L2* as a germline predisposition gene may inform personalized immunotherapy in lymphoma patients.

## TRANSPARENT PEER REVIEW

The peer review history for this article is available at https://publons.com/publon/10.1002/hon.3033.

TO THE EDITOR

1

Family segregation of lymphoma has been long observed. First‐degree relatives of a patient with a lymphoid malignancy have a 1.8‐fold, 1.2‐7‐fold, and 9.8‐fold elevated risk of developing non‐Hodgkin's lymphoma, Hodgkin's lymphoma (HL), and diffuse large B cell lymphoma (DLBCL), respectively.^1^ Even seemingly sporadic cases of lymphoma can have a heritable component and next‐generation sequencing has facilitated the identification of dozens of genes as contributors to germline predisposition to lymphoid malignancies.^1^

Identifying genetic predispositions can both dramatically impact patient management and significantly benefit at‐risk family members. In this regard, the World Health Organization has included germline predisposition to myeloid malignancies in its updated classification guidelines and recommends that the presence of specific underlying genetic defects and predisposition syndrome should be noted as part of diagnosis.^2^

**FIGURE 1:**
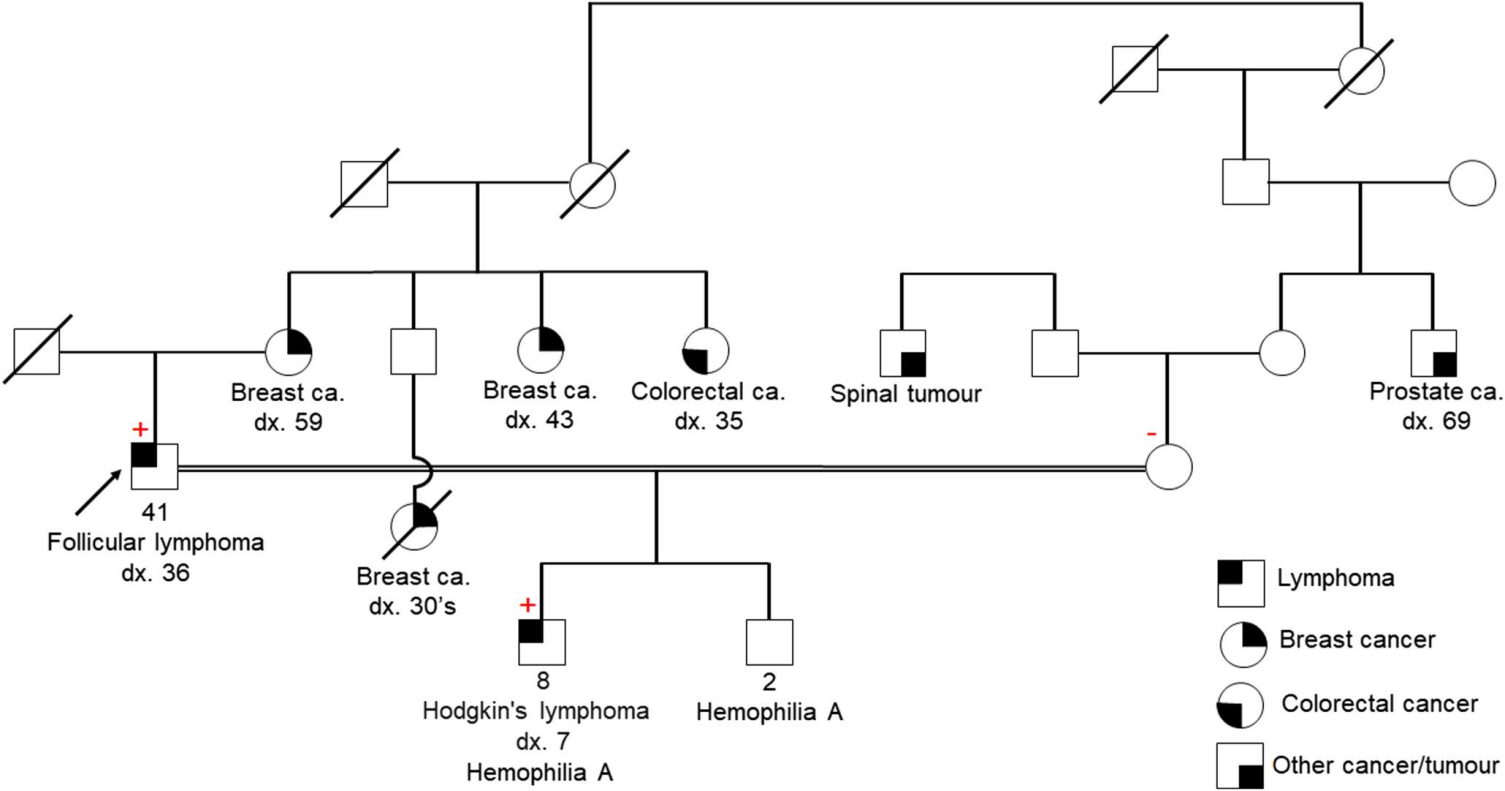
Pedigree of the family with the *PD‐L2* novel variant. Arrow indicates the proband. +: positive for *PD‐L2* c.817‐1G>T variant; ‐: negative for *PD‐L2* c.817‐1G>T variant. Dx: diagnosis

**FIGURE 2:**
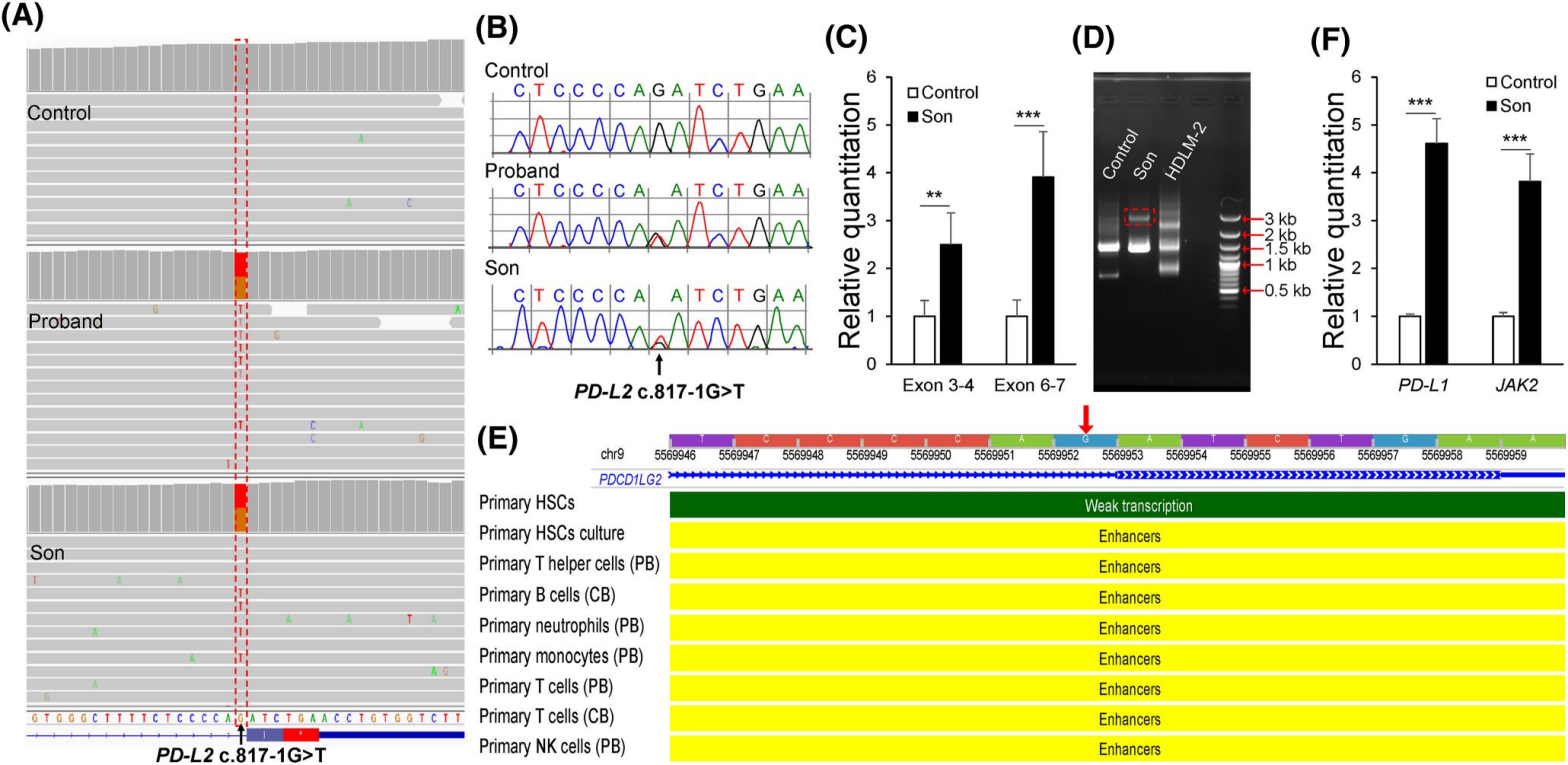
*PD‐L2* alterations identified in patients. *PD‐L2* c.817‐1G>T variant identified by whole‐exome sequencing (WES) shown in Integrated Genomics Viewer (A) and by Sanger sequencing for confirmation (B). (C) RNA expression of *PD‐L2* in the son compared to the control. One pair of primers was designed on exons 3 and 4; the other was designed on exons 6 and 7. Two‐tailed *p* values were calculated between the son and the control from three independent experiments using Student's *t*‐test. **, *p* < 0.01; ***, *p* < 0.001. (D), Electrophoresis of 3′ RACE polymerase chain reaction product. HDLM‐2, a cell line with high expression of *PD‐L2*, was used as a control. The fragment with a box with red dashed lines was from the abnormal *PD‐L2* transcript. (E) *PD‐L2* variant is located in a putative enhancer predicted by Roadmap Epigenomics Project. Data visualization was performed using WashU Epigenome Browser. CB: cord blood. HSC: hematopoietic stem cells. PB: peripheral blood. (F) RNA expression of *PD‐L1* and *JAK2* in the son compared to the control. Two‐tailed *p* values were calculated between the son and the control from three independent experiments using Student's *t*‐test. ***, *p* < 0.001

Despite recent progress, additional cancer predisposition genes remain to be discovered. To reveal novel genetic causes to lymphoma, we performed whole‐exome sequencing in families with a strong history of lymphoma but with no pathogenic/likely pathogenic variants identified during clinical gene panel testing. We discovered a novel variant in the programmed cell death 1 ligand 2 gene (*PD‐L2*, also known as *B7‐DC*, *CD273*, or *PDCD1LG2*) in one family with early‐onset lymphomas and other cancers (Figure 1). The patient (proband) was diagnosed with follicular lymphoma at age 36 and his son was diagnosed with HL at age 7. The proband's mother, two maternal aunts and other family members were diagnosed with breast or colorectal cancer at young age (Figure 1). Clinical hereditary lymphoma gene panel testing of 22 known lymphoma predisposition genes (Table S1) did not reveal any pathogenic or likely pathogenic variants in the proband. Whole‐exome sequencing on the blood of the proband and his son identified a novel variant (NM_025239.4: c.817‐1G>T) in *PD‐L2* (Figure 2A) in them both. The variant was not present in the proband's consanguineous partner who was asymptomatic at evaluation (Figure 2B). The c.817‐1G>T variant affects the canonical splice acceptor site of the intron 6, the last intron of the *PD‐L2* gene. Splicing prediction tools, such as Human Splicing Finder and MaxEntScan, predict that the sequence change dramatically disrupts the normal splicing site. To the best of our knowledge, it has not been described in general population databases, such as the Genome Aggregation Database (gnomAD), or reported as a pathogenic variant in any patients.

PD‐L2 is the second ligand for programmed cell death protein 1 (PD‐1) and is expressed on B cells, dendritic cells, macrophages, activated CD4 and CD8 T cells, lymphomas, and solid tumors.^3^ *PD‐L2* is adjacent to programmed cell death 1 ligand (*PD‐L1*, *CD274*) and Janus kinase 2 (*JAK2*) on chromosome 9. Genomic alterations, such as copy number gain, amplification, rearrangements, and polysomy of chromosome 9p24.1 lead to increased expression of *JAK2*, *PD‐L1*, and *PD‐L2* in a broad spectrum of lymphoid malignancies and solid tumors.^3^ In classic HL, up to 97% of patients have *JAK2/PD‐L1/PD‐L2* locus alterations, which activate JAK2‐STAT signaling and are associated with significantly shorter progression‐free survival.^4^

We evaluated *PD‐L2* expression in blood in the proband's affected son and unaffected partner whose blood samples were available for this study. We found that *PD‐L2* RNA expression was significantly increased in the affected son compared to the partner, who was used as a control (Figure 2C). To investigate the underlying mechanism, we first performed reverse transcription polymerase chain reaction (PCR) and Sanger sequencing using multiple sets of primers alongside the variant, but no abnormal alternative splicing was observed (data not shown). Because sequence changes of the terminal splice acceptor can impair 3'end cleavage at the poly(A) site and abolish transcriptional termination,^5^ we performed 3′ rapid amplification of cDNA ends (3′ RACE). Using gene‐specific and oligo (dT) primers, we observed an additional RNA fragment with a larger size (∼3kb) than a normal 3′untranslated region, (∼1.3kb) in the son (Figure 2D). Sanger sequencing of the PCR fragments confirmed a *PD‐L2* origin. This data show that the c.817‐1G>T change may disrupt the reciprocal functional coupling between the terminal splice acceptor site and the poly(A) signal, resulting in an abnormally elongated *PD‐L2* transcript. As genetic alterations of chromosome 9p24.1 are common in lymphoma, we analyzed copy number variation in the *PD‐L2* locus. We did not find any DNA amplification or chromosomal rearrangement in this region in the proband or his affected son (Figure S1). Therefore, the increased *PD‐L2* RNA expression may result from the elongated *PD‐L2* transcript. The role of the abnormal *PD‐L2* transcript in cell immune function and tumorigenesis needs further investigation.

The *PD‐L2* c.817‐1G is also embedded in a putative enhancer that was demonstrated by chromatin state study in human primary blood cells (Figure 2E).^6^, ^7^ The putative enhancer was shown to interact with promoters of *PD‐L1* and *JAK2* in blood cells of multiple lineages (Figure S2).^8^ Therefore, we investigated *PD‐L1* and *JAK2* RNA expression and found that both were significantly upregulated in the proband's affected son compared to the control (Figure 2F). The c.817‐1G>T variant may increase the activity of the enhancer and lead to increased expression of *PD‐L1* and *JAK2*, although additional study will be necessary to confirm this hypothesis.

To date, over 400 variants in the coding regions of *PD‐L2* have been described in tumors in the Catalog of Somatic Mutations in Cancer database. However, the clinical significance of these variants is not well characterized. In tumors, PD‐L1 and PD‐L2 bind to PD‐1 expressed on activated T cells, attenuate or arrest T cell receptor‐associated downstream signaling, downregulate cytokine production, and inhibit T‐cell proliferation.^3^ Notwithstanding binding to PD‐1, PD‐L2 also binds the second receptor repulsive guidance molecule b to regulate the development of respiratory tolerance.^3^ Although PD‐L2 is well known for immune regulation, a recent study reported that *PD‐L2* functions as an oncogene in osteosarcoma via *RhoA‐ROCK‐LIMK2* and autophagy pathways.^9^ Mutations or aberrant expression of *PD‐L2* may contribute to tumorigenesis through one or more of the above mechanisms. The contribution of *PD‐L2* c.817‐1G>T to the lymphomas in the family is likely twofold. First, c.817‐1G>T results in an elongated *PD‐L2* transcript with increased *PD‐L2* expression, that is, gain of function. Second, it may lead to increased *PD‐L1* and *JAK2* enhancer activity independent of any effect on *PD‐L2* transcription, thereby activating the JAK2‐STAT signaling pathway.

Blocking the interaction of PD‐L1/PD‐L2 and PD‐1 using immune checkpoint inhibitors (ICIs), such as nivolumab or pembrolizumab, is a recent revolution in treating lymphoma and many other cancers.^3^ Although PD‐L1 expression is widely used as a predictive biomarker for ICI treatment, recent studies suggest that PD‐L2 expression can be detected in the absence of PD‐L1 overexpression in some tumor types and may indicate significant clinical predictive response to ICIs independent of PD‐L1 expression.^3^ Higher PD‐L2 expression in tumor and immune cells has also been demonstrated in association with high immune infiltration and improved outcomes in follicular lymphoma and DLBCL.^3^, ^10^ Therefore, patients with elevated *PD‐L2* expression resulting from sequence changes may benefit from ICI therapy. Identifying germline pathogenic variants in *PD‐L2* may inform personalized immunotherapy in patients.

To our knowledge, this is the first report of *PD‐L2* germline variant as a potential genetic predisposition to lymphoma. As this is a single‐family study, evaluation of additional patients and families will improve our understanding of the prevalence of germline *PD‐L2* pathogenic variants in lymphomas. Other family members in our study who were diagnosed with breast and colorectal cancers were not available for further investigation. Whether defects in *PD‐L2* genetically predispose to other cancers is an interesting question to explore. Comprehensive functional studies of *PD‐L2* will delineate the underlying mechanisms and provide evidence for variant interpretation and clinical management of lymphoma patients.

In summary, our study revealed a *PD‐L2* variant as a potential cause of germline predisposition to lymphoma. The novel *PD‐L2* variant may lead to overexpression of *PD‐L2*, *PD‐L1*, and *JAK2* and likely underlies the genetic etiology of the lymphomas in the family. Our study also revealed two uncommon mechanisms of mutation impact: 1) a variant affecting the terminal canonical splice acceptor site can disrupt 3′ end processing of RNA, resulting in an abnormal transcript with increased gene expression; 2) a variant in a transcription regulatory element can alter expression of other genes. Our findings suggest a role of *PD‐L2* in predisposition to lymphoma and provide a potential therapeutic marker for lymphoma treatment.

## CONFLICT OF INTEREST

The authors declare no conflict of interest.

## FUNDING INFORMATION

10.13039/100000048; American Cancer Society; RSG‐17‐044‐01‐LIB

## ETHICS STATEMENT

This study was approved by the institutional review board at the University of Chicago. All individuals signed informed consent forms to participate in the study.

## AUTHOR CONTRIBUTIONS

Jianming Shao, Lei Gao, and Marco L. Leung performed the experiments. Jianming Shao, Lei Gao, Marco L. Leung, Bailey Gallinger, Cara Inglese, M. Stephen Meyn, Daniela Del Gaudio, Soma Das, and Zejuan Li analyzed and interpreted the data. Jianming Shao and Zejuan Li wrote the paper. Zejuan Li designed and coordinated the study.

## Supporting information

Supporting Information S1

## Data Availability

The data that support the findings of this study are available on request from the corresponding author. The data are not publicly available due to privacy or ethical restrictions.
