# Supplementary material for: Exome sequencing identifies PD‐L2 as a potential predisposition gene for lymphoma
Source: Hematol Oncol. 2022 May 29;40(3):475–8. doi: 10.1002/hon.3033 (PMC9546357; doi:10.1002/hon.3033)
Supplement: Supplementary file 1 — Supporting Information S1 [file HON-40-475-s001.docx]

**Exome sequencing identifies *PD-L2* as a potential predisposition gene for lymphoma**

Jianming Shao, Lei Gao, Marco Leung, Bailey Gallinger, Cara Inglese, M. Stephen Meyn, Daniela Del Gaudio, Soma Das, Zejuan Li

**Supplemental Information**

**Materials and methods**

**DNA and RNA extraction and RNA expression analysis**

Genomic DNA was isolated from blood leukocytes on the AutoGenFlex STAR robotic workstation (Autogen, Inc. Holliston, MA) following manufacturer's instructions. Total RNA was isolated from peripheral blood using the PAXgene PreAnalytiX kit (Qiagen, Germantown, MD). A real-time reverse transcription-polymerase chain reaction (RT-PCR) assay was performed using PowerUp SYBR Green Master Mix (ThermoFisher) on the StepOne Real-Time PCR System (Applied Biosystems, Foster City, CA). Primer sequences for *PD-L2*, *JAK2,* and *PD-L1* RNA expression are listed in Table S2.

**Whole-exome sequencing**

Whole-exome sequencing was performed as previously described.^1^ Briefly, targeted germline genomic DNA fragments were obtained by shearing genomic DNA, repairing the ends of the sheared DNA, and ligating index-specific paired-end adaptors. DNA fragments were hybridized to Agilent SureSelect Clinical Research Exome probes (Agilent Technologies, Santa Clara, CA) for target enrichment. Hybridized molecules were amplified using universal PCR primers. DNA libraries were normalized, and five samples were pooled in one run and sequenced using standard Illumina paired-end primers and chemistry on the Illumina NextSeq 500 sequencer (Illumina, San Diego, CA).

**Whole-exome sequencing data analysis**

Data were analyzed using a custom bioinformatics pipeline developed from publicly available bioinformatics tools. Briefly, the quality and integrity of sequence data were assessed using FastQC. Raw data were aligned to the reference human genome hg19 using BWA. Reads in certain problematic areas were identified and realigned using GATK. The depth and completeness of the data set, defined as alignment statistics, were generated using GATK. Variants were called on the final alignment file using GATK’s UnifiedGenotyper tool. Raw variants were further filtered using GATK’s best practice guidelines to retain high-quality variants that were then annotated using the Alamut–Batch software and further filtered based on the global population frequency in gnomAD. The final list of variants with minor allele frequency lower than 1% were deposited into a MySQL database for further analysis. As we assumed affected individuals shared a common genetic defect, we analyzed variants shared by affected family members. The copy number variation (CNV) of patients was analyzed using ExomeDepth and the reference was developed from eight exome sequencing samples.

**3’ Rapid amplification of cDNA ends (RACE)**

We first performed touchdown PCR using RACE primer set 1 (Table S2) and QIAGEN OneStep RT-PCR Kit (Qiagen, Germantown, MD). The touchdown PCR condition is shown in Table S3. Then, we performed a nest PCR with 20 cycles using 1 µL of the first PCR product, RACE primer set 2 (Table S2), and KAPA HiFi HotStart ReadyMix PCR Kit (Roche Diagnostics, Indianapolis, IN). The nest PCR product was purified using Zymoclean Gel DNA Recovery Kit (Zymo Research, Irvine, CA) and Sanger sequenced by GENEWIZ (South Plainfield, NJ) to confirm the origin.

***PD-L2* putative enhancer visualization and statistical analyses**

Chromatin status data was downloaded from Roadmap Epigenomics Project.^2^ Interaction of the putative enhancer with promoters of *PD-L1* and *JAK2* was reported in a promoter capture Hi-C study.^3^ Visualizations were performed using WashU Epigenome Browser.^4^ The statistical significance of RNA expression differences between patient and control was determined using a Student's t-test.

**Ethics approval and consent to participate**

This study was approved by the institutional review board at the University of Chicago. The patients or the parents signed informed consent forms to participate in the study.

**Table S1. Hereditary lymphoma panel gene list.**

| **Tier 1 Hereditary Lymphoma Panel** | | | | | | | |
| --- | --- | --- | --- | --- | --- | --- | --- |
| CHEK2 | KLHDC8B | MLH1 | MSH2 | MSH6 | NPAT | PMS2 | TP53 |
| **Tier 2 Hereditary Lymphoma Panel** | | | | | | | |
| *ADA* | *ATM* | *BLM* | *BRCA1* | *BRCA2* | *CARD11* | *FAS* | *NBN* |
| *NF1* | *PRF1* | *SH2D1A* | *STXBP2* | *TNFRSF13B* | *WAS* |  |  |

**Table S2. Primer sequences.**

| **Primer name** | **Forward primer** | **Reverse primer** |
| --- | --- | --- |
| *PD-L2* exon 3-4 | CTCGTTCCACATACCTCAAGTCC | CTGGAACCTTTAGGATGTGAGTG |
| *PD-L2* exon 6-7 | ACCTGTCACCACAACAAAGAG | GCCACCGAATTCTTGTTCAG |
| *PD-L1* | AAAAATGTGGCATCCAAGATACA | CACAGGTTGAGAATCCCTGC |
| *JAK2* | GTCATGTCTTACCTCTTTGCTC | TCATCATATCTAACACTGCCATCC |
| *GAPDH* | CCTCCCGCTTCGCTCTCT | CTGGCGACGCAAAAGAAGAT |
| RACE primer set 1 | ACCTGTCACCACAACAAAGAG | CCAGTGAGCAGAGTGACGAGGACTCGAGCTCAAGCTTTTTTTTTTTTTTTTT |
| RACE primer set 2 | AAAGAGGGAAGTGAACAGTGC | GAGGACTCGAGCTCAAGC |

**Table S3. The condition of thermal cycling for touchdown PCR.**

| Temperature | Time |  | Cycles |
| --- | --- | --- | --- |
| 50 °C | 30 min |  | 1 |
| 95 °C | 15 min |  | 1 |
| 94 °C | 30 s |  | 18 |
| 68-50 °C (-1°C/cycle) | 30 s |  |  |
| 68 °C | 5 min |  |  |
| 94 °C | 30 s |  | 22 |
| 60 °C | 30 s |  |  |
| 68 °C | 5 min |  |  |
| 68 °C | 10 min |  | 1 |

**
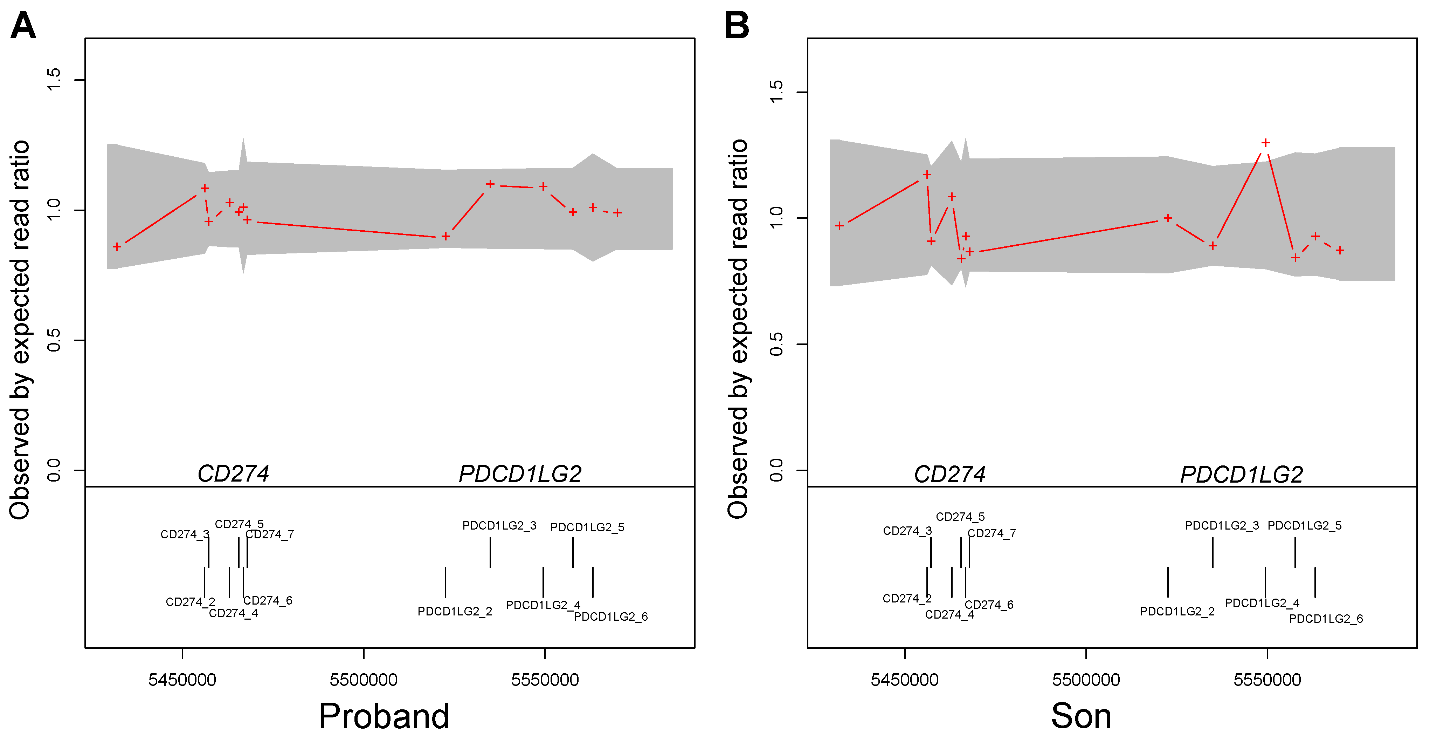
Figure S1.** Analysis of copy number variation in the *PD-L1* (*CD274*) and *PD-L2* (*PDCD1LG2*) locus in the proband (**A**) and son (**B**). Red crosses show the ratio of observed to expected number of reads in the patient sample. The grey shaded region shows the estimated 99% confidence interval for this observed ratio in the absence of CNV call.


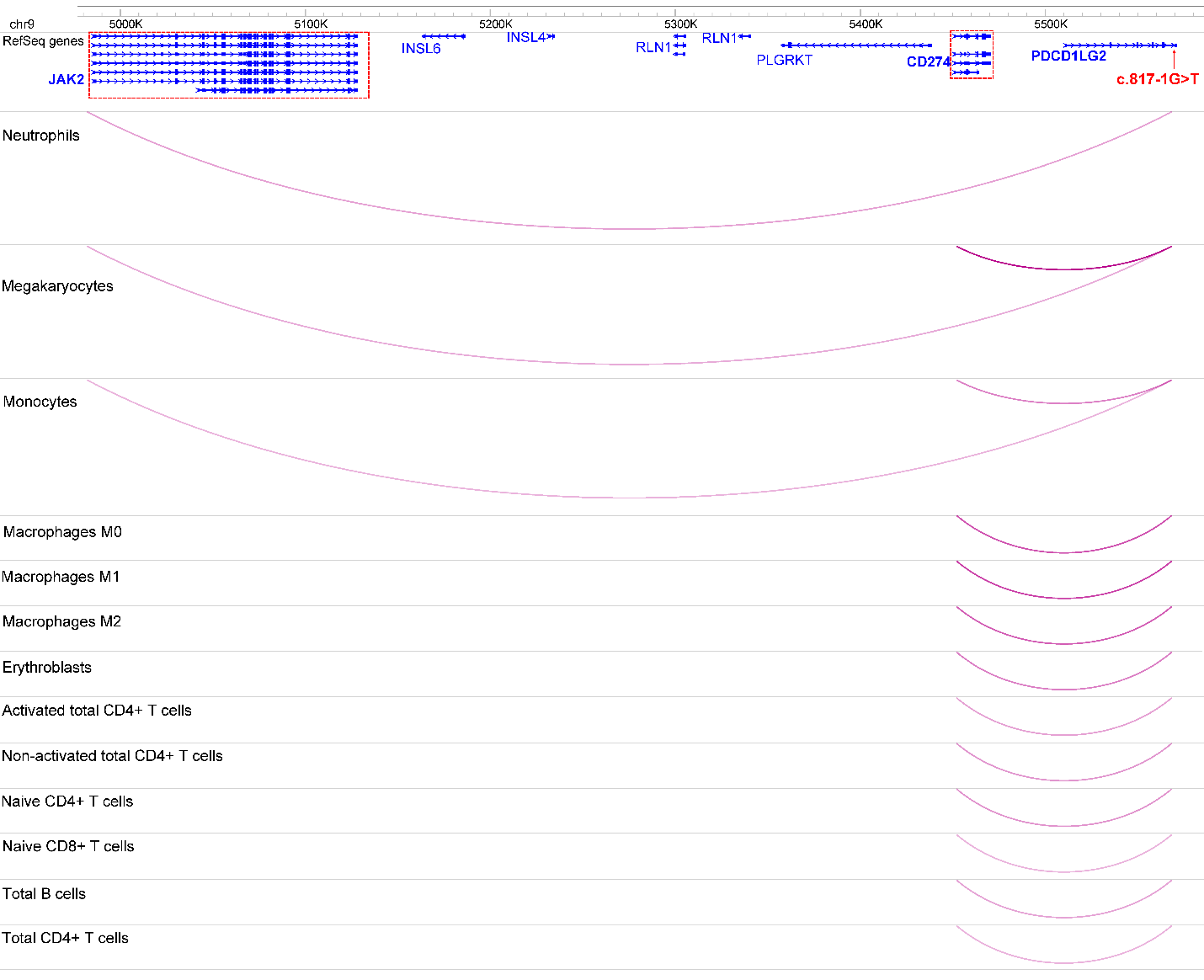


**Figure S2.** Interaction of the putative enhancer within the region of c.817-1 in *PD-L2* with promoters of *PD-L1* and *JAK2* in blood cells by a capture Hi-C study. Pink lines represent interactions of the two loci.

**References**

1. Sun M, Johnson AK, Nelakuditi V, et al. Targeted exome analysis identifies the genetic basis of disease in over 50% of patients with a wide range of ataxia-related phenotypes. *Genet Med*. Jan 2019;21(1):195-206. doi:10.1038/s41436-018-0007-7

2. Roadmap Epigenomics C, Kundaje A, Meuleman W, et al. Integrative analysis of 111 reference human epigenomes. *Nature*. Feb 19 2015;518(7539):317-30. doi:10.1038/nature14248

3. Javierre BM, Burren OS, Wilder SP, et al. Lineage-Specific Genome Architecture Links Enhancers and Non-coding Disease Variants to Target Gene Promoters. *Cell*. Nov 17 2016;167(5):1369-1384 e19. doi:10.1016/j.cell.2016.09.037

4. Li D, Hsu S, Purushotham D, Sears RL, Wang T. WashU Epigenome Browser update 2019. *Nucleic Acids Res*. Jul 2 2019;47(W1):W158-W165. doi:10.1093/nar/gkz348
